# Fractional Orbital Angular Momentum Sorting Using Wind Spiral Transformation

**DOI:** 10.1002/nap2.70086

**Published:** 2026-04-17

**Authors:** Xiaobin Yang, Mengran Fan, Hao Zhang, Kun Liu, Juntao Hu, Yuping Tai, Yijie Shen, Xinzhong Li

**Affiliations:** ^1^ School of Physics and Engineering & School of Chemistry and Chemical Engineering Henan University of Science and Technology Luoyang China; ^2^ State Key Laboratory of Transient Optics and Photonics Xi'an Institute of Optics and Precision Mechanics, Chinese Academy of Sciences Xi'an China; ^3^ Centre for Disruptive Photonic Technologies, School of Physical and Mathematical Sciences Nanyang Technological University Singapore; ^4^ School of Electrical and Electronic Engineering Nanyang Technological University Singapore

## Abstract

Orbital angular momentum (OAM) mode sorting is a crucial component of the optical multiplexing systems based on the optical vortex beam carrying OAM. However, the number of sortable integer‐order OAM states remains limited. Herein, we propose a general spiral transformation, that is, wind spiral transformation, to conduct sortable fractional‐order OAM states to solve the existing drawback. By transforming a fractional‐order OAM mode into a superposition of integer‐order OAM states, the sorting capacity of OAM mode is significantly extended, where the proposed method is suitable for integer and fractional OAM mode sorting. Further, the proposed scheme is applied to image encoding and decoding, resulting in good image reconstruction and unlocking a scalable paradigm for high‐dimensional information encoding and decoding. This proposed sorting method shows great potential for implementing highly confidential data storage systems, which may encourage further applications in classical and quantum regimes.

## Graphical abstract

A wind‐spiral coordinate transformation enables mode sorting of fractional‐order orbital angular momentum (OAM) states, overcoming the limited channel count of conventional integer only sorters. Fractional OAM is mapped into a controlled superposition of integer modes, producing distinctive multi‐peak signatures for discrimination. The method supports both integer and fractional OAM sorting and is demonstrated in image encoding/decoding, suggesting scalable high‐dimensional information processing.

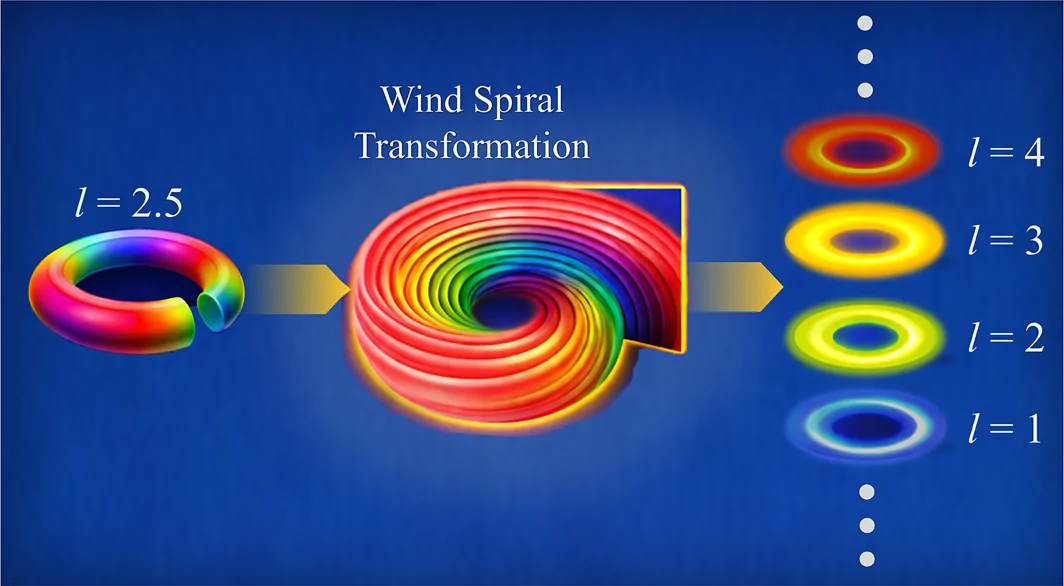

## 1 Introduction

Optical vortex (OV), which possesses a spiral phase structure in the form of exp(i*lθ*), carries an additional degree of freedom, namely, the orbital angular momentum (OAM) [1, 2, 3]. In the spiral phase structure exp(i*lθ*), *θ* is the azimuthal angle around the beam axis, and the integer *l* is the topological charge (TC), where the magnitude of OAM is *l*ℏ per photon [4, 5]. Recently, the OAM in OV beam has attracted significant attention in optical communication [6, 7] and quantum information due to its discrete mutually orthogonal modes in the spatial dimension [8, 9, 10].

There are many methods to generate an OV beam with OAM, such as using q‐plates, spiral phase plates, and integrated on‐chip components [11, 12, 13]. Besides generation, OAM mode sorting is a key issue for optical communication systems, which efficiently decomposes different OAM modes in terms of power loss and intermodal crosstalk [14, 15, 16]. Based on Mach–Zehnder interferometers, the OAM modes are sorted efficiently; however, complexity increases with the number of sorted OAM modes [17]. The same complexity also appeared in the mode sorter based on multi‐plane light conversion [18, 19]. The simplest way to circumvent this complexity was to use a specially designed Dammann grating [20] or cascaded tunable resonator [21], which diffracted different OAM‐states into different diffraction orders. However, the specially designed grating only works for a specific TC, where the efficiency was limited to 1/*N*, where *N* is the number of OAM‐states.

To sort the OAM states efficiently, Berkhout et al. proposed a method based on the log‐pol coordinate transformation [14]. However, mode overlap between adjacent OAM states occurred due to the limitations of the log–pol coordinates transformation in mathematics. Adding a fan‐out element can effectively reduce mode overlap at the expense of device efficiency and simplicity [15]. Yu et al. proposed a logarithmic spiral transformation to achieve low mode overlap without increasing the complexity of the device [22]. Cheng et al. achieved lower mode overlap by replacing the logarithmic with Archimedes’ spirals transformation, whereas the cost is a reduction in the OAM recognition range and the appearance of background noise [23]. Furthermore, coordinate transformation is not only for OAM sorting but also for designing a specific coordinate transformation to realize OAM states multiplication [24, 25, 26, 27]. In summary, it is an effective method to reduce mode crosstalk by optimizing the types of spiral transformation. The references above are focused on sorting integer OAM states. Predictably, fractional OAM states will provide more versatile modes for expanding information capacity.

To achieve fractional OAM state sorting with high resolution, we propose a general spiral model and apply it in coordinate transformation, that is, wind spiral transformation, which may be used to realize efficient and low‐crosstalk OAM mode sorting. The key idea is to increase the number of spiral turns within the same annular width. Compared with the existing spiral transformations, the proposed method exhibits more helical turns within the same annular width. Because the width of the characteristic peak is inversely proportional to the number of helical turns, the proposed wind spiral transformation helps to reduce mode crosstalk. Consequently, it offers a promising approach for efficiently sorting fractional‐order OAM states. In our case, the Perfect Laguerre–Gaussian (PLG) beam is chosen as the carrier of integer and fractional OAM states, owing to its minimal variation in radius with different fractional TCs. The experimental results also show that the proposed wind spiral transformation has optimistic performance in integer or fractional OAM states sorting. Further, the wind spiral transformation is applied in image encoding and decoding, which provides an approach to similar research areas such as information encryption, ultrahigh‐speed photography, and high‐dimensional information multiplexing with coordinate transformation [28, 29, 30, 31].

## 2 Principles, Methods, and Experimental Setup

As mentioned above, OAM mode crosstalk negatively correlates with the number of spiral turns. Increasing spiral turns is beneficial for reducing the mode crosstalk. Inspired by the trajectory of an aircraft, when a plane flying at a speed of ***v***_**1**_ encounters a ground wind with speed ***v***_**2**_, its resultant velocity becomes ***v***_**3**_ = ***v***_**1**_ + ***v***_**2,**_ as shown in the inset of Figure 1. (More details see Supporting Information S1: Note 1). Compared with the logarithmic spiral used in traditional spiral transformation, the wind spirals have more turns under the same OV size condition. To highlight the main idea, we compare the principal schemes between the logarithmic spiral transformation of Figure 1a and the proposed wind spiral transformation of Figure 1b. Because the logarithmic spiral contains an exponential function, the spiral radius *r* shows steep variation with the increase of *θ*. As illustrated in Figure 1a, for an input OAM state with topological charge *l*, the 2π*l* excursion of the phase is transformed into a stripe‐shaped output beam with the same phase distribution in the output plane. After passing through the lens, the input OAM states are sorted into different positions in the focal plane. As shown in Figure 1b, this proposed wind spiral transformation works by modulating the stripe‐shaped output beam to carry multiple copies of the 2π*l* phase excursion, thereby reducing mode crosstalk and achieving fractional OAM states sorting.

**FIGURE 1:**
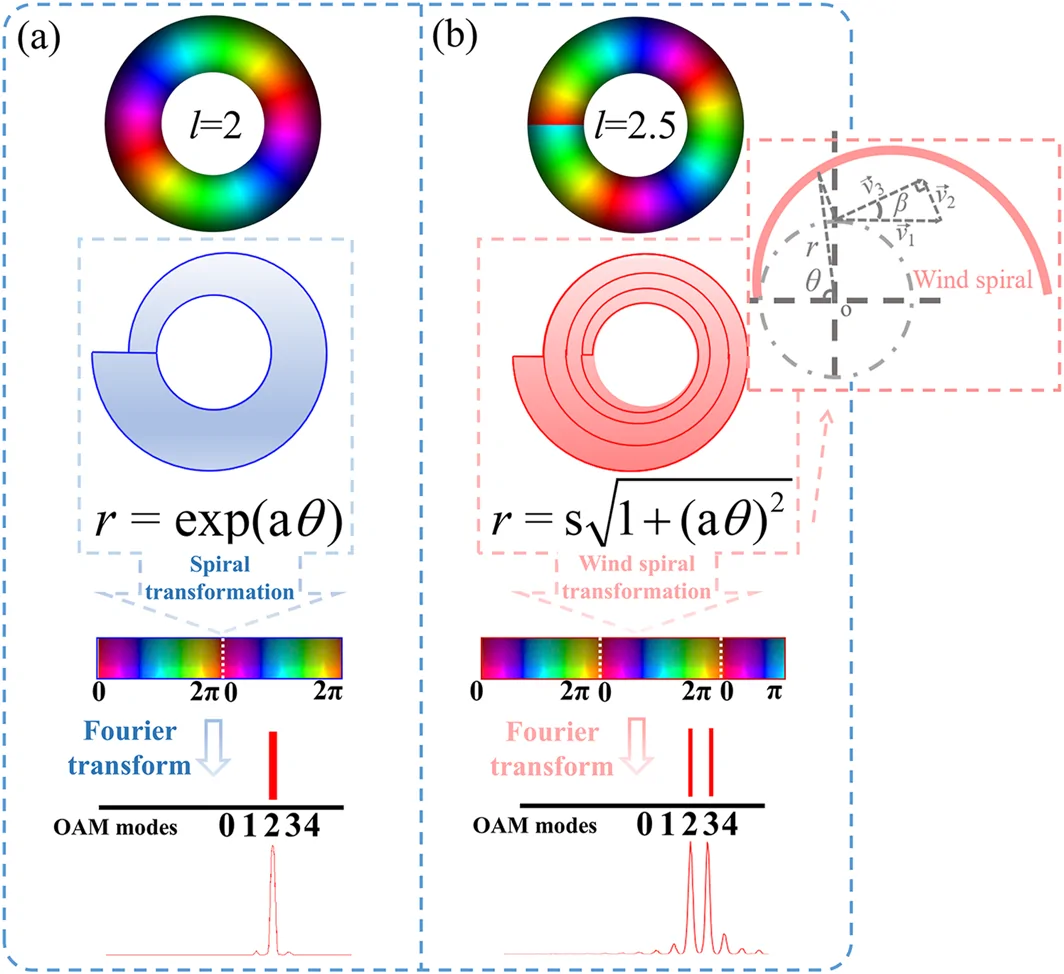
Principle comparison between the schemes of the typical traditional spiral transformation (a) and the proposed wind spiral transformation (b) for OAM mode states sorting. The inset is a conceptual diagram of the wind spiral, which is inspired by the trajectory of an aircraft.

Under the paraxial approximation, and assuming a propagation distance *d* between the input plane (*x*, *y*) and the output plane (*u*, *v*), as shown in Figure 2a, the phase profile required to achieve the optical mapping between the two planes can be derived from geometrical optics [32]. The spiral transformation formula is given by the following equation (for a more detailed process, see Supporting Information S1: Note 2):

(1) $$Q_{x}=k\frac{u-x}{d},Q_{y}=k\frac{v-y}{d},$$

where *Q* represents the phase modulation applied on the input plane (also referred to as the spiral transformation plane) to implement the desired coordinate transformation. The terms *Q*_*x*_ and *Q*_*y*_ represent the partial derivatives of *Q* with respect to *x* and *y*, respectively. *k* = 2π/λ denotes the wave number. (*x*, *y*) and (*u*, *v*) denote the spatial coordinates on the input and output planes. According to the spiral coordinate transformation in geometric optics [32], the expression of *Q* is satisfied by the following necessary and sufficient conditions,

(2) $$\left\{\begin{array}{l} Q_{x}=\frac{k\beta }{d}\theta -\frac{kx}{d}\\ Q_{y}=\frac{k\beta }{d}\left[\ln\left(\frac{r}{r_{0}}\right)\right]-\frac{ky}{d} \end{array}\right..$$

**FIGURE 2:**
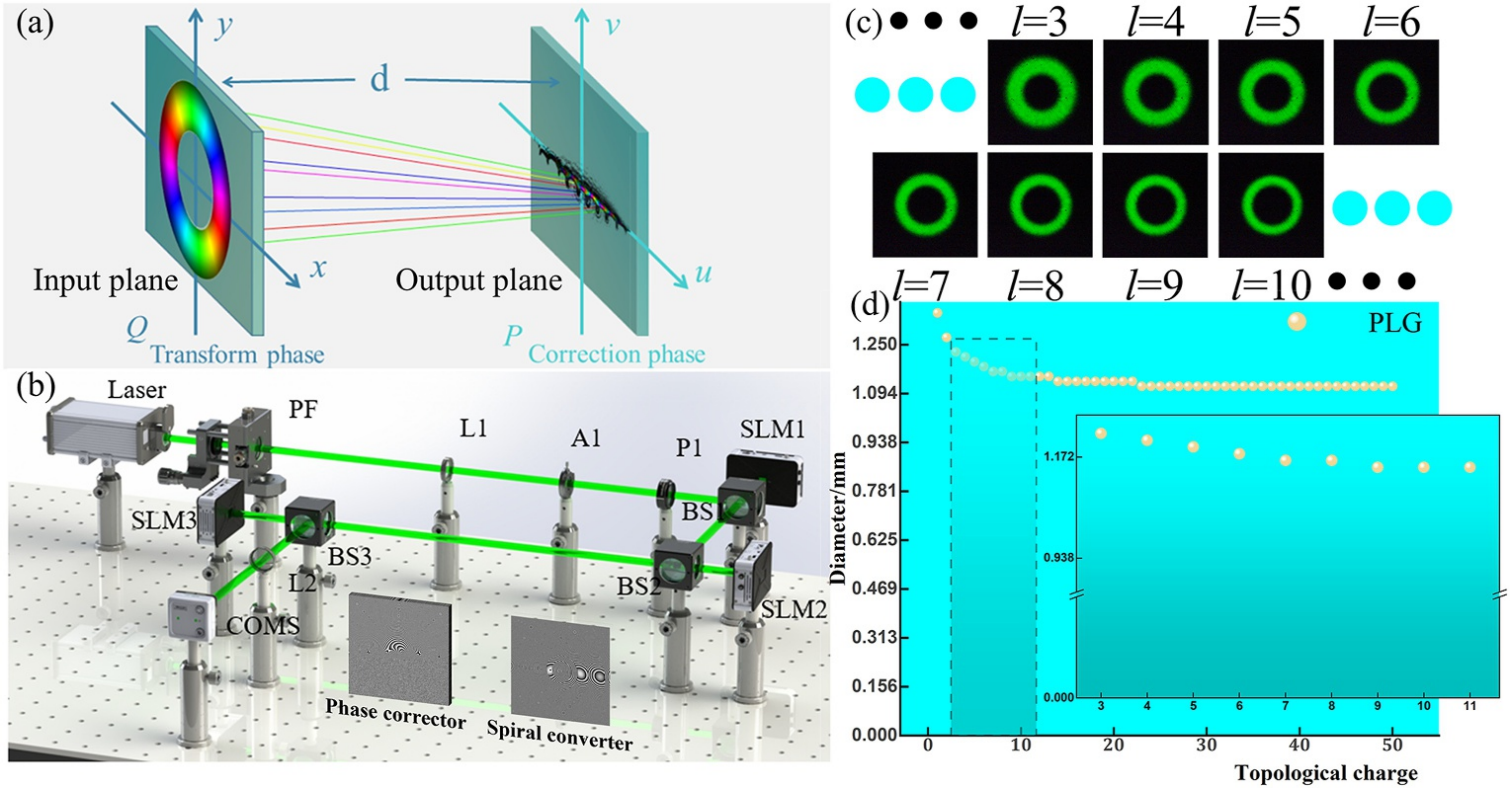
(a) Schematic diagram of the wind spiral transformation. (b) Experimental setup for spiral transformation and OAM sorting. PF: pinhole filter. L1 and L2: lenses. A1 and P1: aperture and polarizer. BS1, BS2, and BS3: beam splitters. SLM1, SLM2, and SLM3: spatial light modulators. CMOS: complementary metal–oxide semiconductor. Insets: Spiral converter and Phase corrector are loaded on SLM2 and SLM3, respectively. (c) Experimental intensity of the PLG beam. (d) PLG beam diameter VS TC.

Then, solving the system of partial derivatives equations, the analytical solution of *Q* is as follows:

(3) $$Q=\frac{k\beta }{d}\left[y\;\ln\left(\frac{r}{r_{0}}\right)+x\theta -y\right]-\frac{k{\left(x^{2}+y^{2}\right)}^{2}}{2d}.$$

To achieve the desired coordinate mapping, the type of spiral is defined as a wind spiral, which is expressed in polar coordinates as *r* = *s*[1 + (a*θ*)^2^]^1/2^, where *r*(*θ* = 0) = *r*_0_, *β* is a scaling parameter that governs the rate of radial expansion. However, this transformation introduces local angular tilts and nonparaxial components, causing the output beam to deviate from the optical axis. The corrective phase term *P*(*u*, *v*) must be introduced to correct these deviations and restore paraxiality. The following relation can be obtained by inversely deriving the relationship between the spiral parameters and coordinate (*u*, *v*), and substituting it into the necessary and sufficient condition for the analytical solution of *P*.

(4) $$\left\{\begin{array}{l} P_{u}=\frac{k}{d}\left[r_{0}\;\exp\left(\frac{v}{\beta }\right)\cos\left(\frac{u}{\beta }\right)-u\right]\\ P_{v}=\frac{k}{d}\left[r_{0}\;\exp\left(\frac{v}{\beta }\right)\sin\left(\frac{u}{\beta }\right)-v\right] \end{array}\right..$$

*P*_*x*_ and *P*_*y*_ are the partial derivatives of *P* with respect to *x* and *y*, respectively. Then, solving the system of partial derivatives equations, the analytical solution of *P* is as follows:

(5) $$P=\frac{k\beta r_{0}}{d}\exp\left(\frac{v}{\beta }\right)\sin\left(\frac{u}{\beta }\right)-\frac{k\left(u^{2}+v^{2}\right)}{2d}.$$

Up to now, the expressions for the two crucial components of the OAM sorting system have been generated, namely the spiral converter and the phase corrector, corresponding to Equations (3) and (5). PLG beam with fractional OAM states was generated, and its complex amplitude was as follows [33]:

(6) $$E_{\text{PLG}}=A_{0}{\left(\frac{\sqrt{2}r}{\omega_{k}}\right)}^{\left|l\right|}L_{n}^{\left|l\right|}\left(\frac{2r^{2}}{\omega_{k}^{2}}\right)\exp\left[-\left(\frac{r^{2}}{\omega_{k}^{2}}+il\theta \right)\right],$$

where *ω*_*k*_ = *ω*(*z*)/2(2*N*)^1/2^, *ω*(*z*) = *ω*_0_[1 + (*z*/*L*_*D*_)^2^]^1/2^, *L*_*D*_ = *k* $\omega_{0}^{2}$/2, and *k* = 2π/λ. $L\begin{array}{l} \left|l\right|\\ n \end{array}$(…) represented Laguerre's polynomial, and *l* and *n* represent the TC of the PLG beam and the radial index, respectively.

To verify the proposed OAM states sorting method, we set up an experimental device as shown in Figure 2b. The laser beam was transformed into an approximate parallel beam by passing through a pinhole filter and lens L1 (*f*_1_ = 150 mm). Apertures A1 and P1 were used to intercept the central part of the expanded beam to obtain an approximate flat–top beam with more even intensity distribution and ensure horizontally polarized light, respectively. The expanded beam passed through the beam splitter BS and illuminated the spatial light modulator 1(SLM1, HOLOEYE, PLUTO‐VIS‐016, pixel size: 8 × 8 μm^2^), which was used to generate PLG beams carrying different OAM states. The modulated beam illuminated at SLM2 (SLM2, CAS MICROSTAR, FSLM‐2K39‐P, pixel size: 4.5 × 4.5 μm^2^) is loaded with a spiral converter, enabling it to transform gradually into a straight line‐shaped beam during propagation. Then, the straightened beam, modulated by the spiral converter, is incident on SLM3 (CAS MICROSTAR, FSLM‐2K39‐P, pixel size: 4.5 × 4.5 μm^2^) loaded with a phase correction mask, transforming into a tilted plane wave. Finally, a Fourier transformation was performed using lens L2 (*f*_2_ = 500 mm), enabling the beams carrying different OAM states to be spatially separated and recorded by a complementary metal‐oxide semiconductor (CMOS, Basler acA1600‐60gc, pixel size of 4.5 × 4.5 μm^2^).

To study the variation of PLG beam diameter with different TCs, the PLG beam and its diameter VS TC of 0–50 were generated and determined, which is depicted in Figure 2c,d. As illustrated in Figure 2d, the PLG beam diameter exhibits a weakly decreasing trend with TC increasing. This property facilitates efficient sorting of OAM states. The comparison between the wind spiral coordinate transformation and the other coordinate transformation is shown in Figure 3. Here, *θ*_max_ denotes the azimuthal angle when the spiral radius reaches *r*_max_, and *N*_period_ _=_ *θ*_max_/2π represents the number of spiral turns within the same vortex. The comparison results indicate that the wind spiral transformation can generate more turns under the same conditions. To quantitatively evaluate and compare the sorting capabilities of different spiral transformations, we introduced a common figure of merit, *Optical Fidelity*, which characterizes the sorting capability of the sorter. *Optical Fidelity* is the ratio of the distance between adjacent OAM states over their average FWHM [34]. In our case, the *Optical Fidelity* achieved by the wind spiral transformation is improved by 45% and 25% compared with the Archimedean spiral and logarithmic spiral transformations, respectively, demonstrating its superior sorting performance. In addition, the mode purity (MP), which reflects the similarity between experimental and simulated results, is evaluated. Due to the presence of background noise, the measured MP is approximately 80%; however, this does not affect the sorting resolution of the sorter.

**FIGURE 3:**
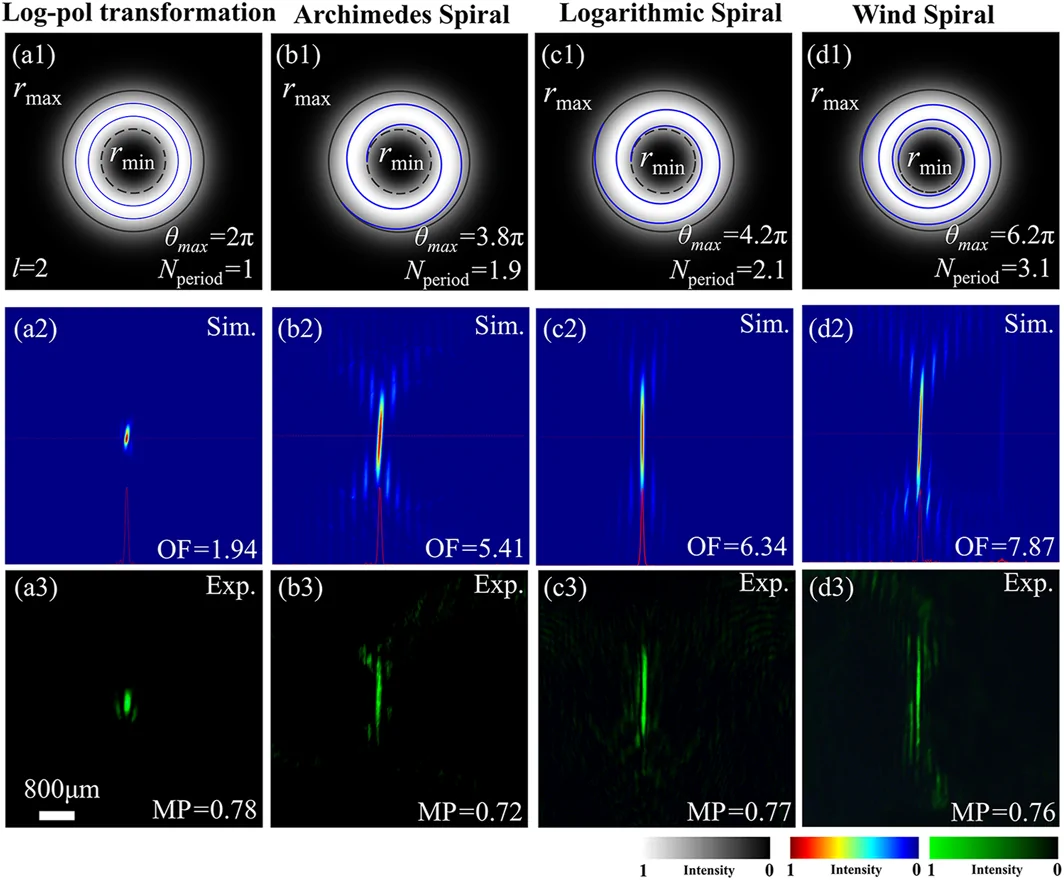
Comparison of different coordinate transformations under the same conditions. (a1) Log‐polar transformation. (b1) Archimedes spiral transformation. (c1) Logarithmic spiral transformation. (d1) Wind spiral transformation. (a2)–(d2) The corresponding sorting results and *optical fidelity* (OF). (a3)–(d3) The experiment results and mode purity (MP).

## 3 Results and Discussions

The integer‐order OAM modes are sorted to validate the sorting capability of the wind spiral transformation. As shown in Figure 4a, input beams are PLG beams with multiple OAM states. After passing through the spiral converter, which is loaded on SLM 2, the intensity is enlarged into a stripe‐shaped beam, as shown in the blue column in Figure 4a, by wind spiral transformation at a propagation distance *d* behind the spiral converter in free space. Next, the light intensity after spiral transformation passes through the phase corrector and lens, resulting in the modulated beam, which is attached to continuous phase and transformed by Fourier transform to generate vertical stripes with different spatial positions, which is called Output, as shown in the yellow column in Figure 4a. The one‐dimensional intensity distribution of the output beam is shown in Figure 4b. Each color represents an incident beam with a distinct TC. The sorting results of PLG beams clearly show characteristic peaks at different horizontal positions, corresponding to different TCs. To quantify the sorting ability of this sorter, the crosstalk is calculated. Because of beam broadening, there is a slight asymmetry between the simulated and experimental results. Notably, the experimental results show inconsistent background noise concerning simulated results. This may be due to SLM phase quantization and imperfect optical systems alignment [35]. However, it does not affect the sorter's sorting ability. In addition, the transverse coordinate of the characteristic peak was the output position. The output position positively correlated with the TC *l*. The relationship is given as follows:

(7) $$\xi_{\left(l\right)}=l\frac{\lambda f_{2}}{2\pi \beta },$$

where *ξ*_(*l*)_ indicates the output transverse coordinate associated with the TC *l. f*_2_ represents the focal length of the lens used for the Fourier transform. In this case of *λ* = 532 nm and *f*_2_ = 500 mm, the separation between adjacent OAM modes is calculated theoretically as *ξ*_(*l*)_ − *ξ*_(*l*‐1)_ = 133 μm. Further, the characteristic peaks were well separated, with the OF calculated as 4.75. The distinct intensity peaks and high optical fidelity provide promising behavior in optical mode sorting, encoding, and decoding.

**FIGURE 4:**
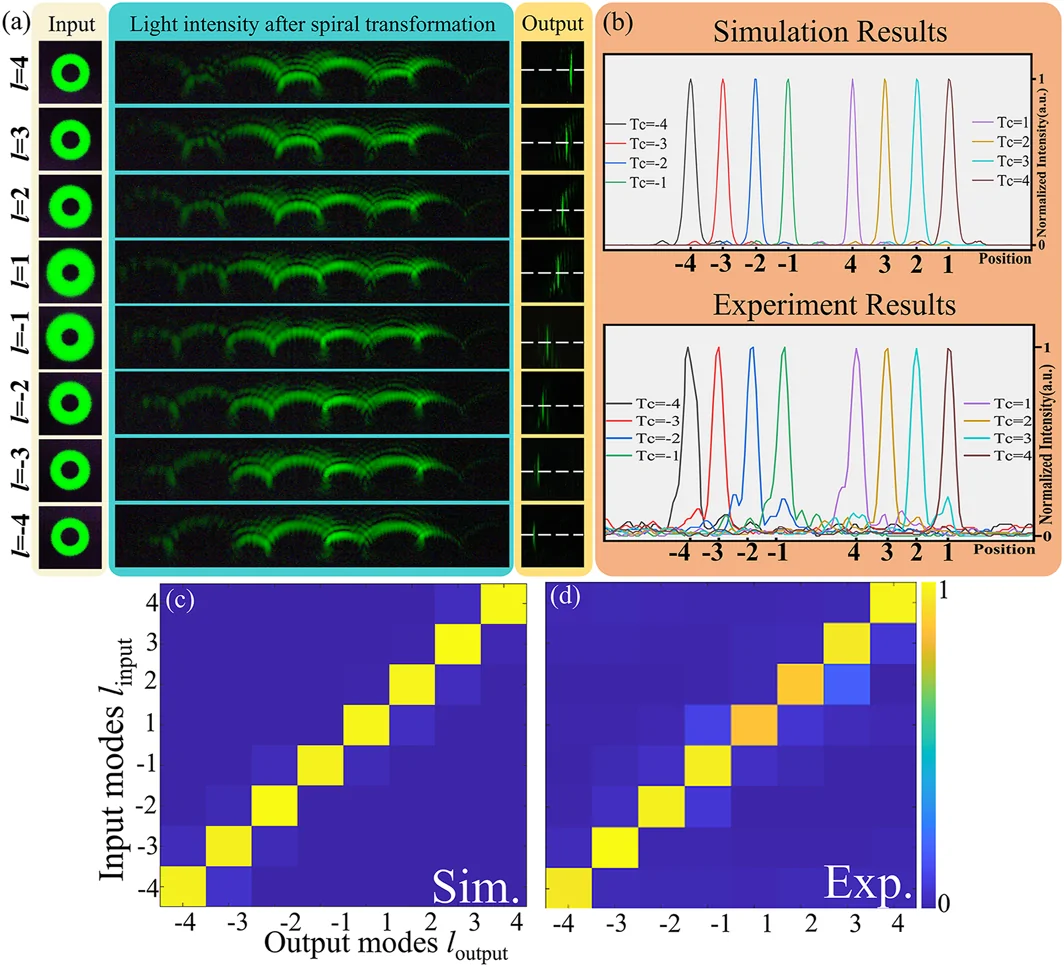
(a) Experimental intensity distribution during the integer OAM states sorting processing in the experiment (More details see Supporting Information S1: Note 3). (b) Simulated and experimental results of the one‐dimensional intensity distribution of the OAM mode sorting. (c) The crosstalk matrix of simulated sorting results. (d) The crosstalk matrix of experimental results. The regions all have the same size and are chosen so that they fill the entire aperture. The intensities are shown as a part of the total intensity of the input beam.

Surprisingly, the wind spiral transformation performs satisfactorily in fractional OAM state sorting, even though it does not uniquely identify the fractional value itself. This property manifested in that fractional OAM states sorting results are overlapping with adjacent integer OAM output. Figure 5a shows that, in contrast to integer OAM sorting results, the intensity patterns after spiral transformation contain dark cores and two bright stripes in the output beams. This is caused by the mismatch in fractional OAM states. Based on the results of theoretical derivation, fractional‐order OAM states fundamentally consist of a coherent superposition of integer‐order OAM states, with the corresponding coefficients governed by a Fourier series distribution. The complex amplitude of the fractional‐order PLG beam is expressed as [36, 37] follows:

(8) $$\begin{array}{l} E_{\text{FPLG}}\left(r,\theta \right)=\sum_{\zeta =-\infty }^{\infty }c_{\zeta }\psi_{n}^{\zeta }\\ c_{\zeta }=\left[1-\exp\left(i2\pi \eta \right)\right]\frac{i\;\exp\left[i\left(l-\zeta \right)\theta_{0}\right]}{2\pi \left(l-\zeta \right)}\exp\left[i\left(l-\zeta \right)\theta \right]\exp\left(-i\eta \theta \right)\\ \psi_{n}^{\zeta }=\frac{C_{\zeta n}}{\sqrt{\omega_{0}}}{\left(\frac{\sqrt{2}r}{\omega_{0}}\right)}^{\left|\zeta \right|}\exp\left(-\frac{r^{2}}{\omega_{0}^{2}}\right)L_{n}^{\left|\zeta \right|}\left(\frac{2r^{2}}{\omega_{0}^{2}}\right)\exp\left(i\zeta \theta \right) \end{array},$$

where *l* = *ζ* + *η*, *ζ* and *η* denote the integer and fractional parts of the topological charge, respectively. *C*_*ξn*_ = {2*n*!/[π(|*ζ*| + *n*)!]}^1/2^ is the normalization coefficient. Analysis shows that dark cores appear in intensity after spiral transformation, as shown in Figure 5a, an enlarged image with a white border, due to partial superposition between two adjacent integer OAM modes. In addition, the two bright stripes in the output are actually formed by the superposition of two adjacent integer OAM states sorting results, as shown in the yellow column in Figure 5a. This also proves the correctness of our analysis on the other side. The OAM state decomposition and the corresponding one‐dimensional intensity distribution are compared. The results, as shown in Figure 5b, demonstrate a high degree of consistency, which provides further evidence for the analysis. To facilitate comparison, we separately plotted the one‐dimensional intensity distributions corresponding to the simulated and experimental results of fractional OAM mode sorting, as shown in Figure 5c. In Figure 5c, the *x*‐axis indicates the position of the characteristic peaks with the left side of the output image as the zero point of the *x*‐axis (in mm); the *y*‐axis shows the normalized 1D intensity distribution; and the *z*‐axis corresponds to incident beams carrying various OAM states. Each color represents a specific OAM state: 3.5, 2.5, 1.5, 0.5, −0.5, −1.5, −2.5, and −3.5, respectively. In comparison with the simulation results, the experimental results display broader characteristic peaks and uneven peak intensities, which are primarily due to unavoidable diffraction and collimation errors. Because of the influence of the two characteristic peaks, FOAM sorting results appear unexpected background noise, which is not conducive to multiplex FOAM (More discussion in Supporting Information S1: Notes 9 and 10). Nevertheless, a high level of agreement was observed between simulation and experimental results. To determine the characteristic peak position of the fractional‐order OAM state, we selected the midpoint between the two intensity peaks as the representative position of the fractional OAM state, as shown in Figure 5d. The horizontal axis denotes the characters represented in decimal form, ranging from 0 to 15, corresponding to the 16 symbols in hexadecimal representation. The vertical axis indicates the characteristic coordinates corresponding to PLG beams with different OAM states. According to the calculation, the Pearson correlation coefficient is 0.99, indicating an excellent agreement between the experimental measurements and the simulation outcomes.

**FIGURE 5:**
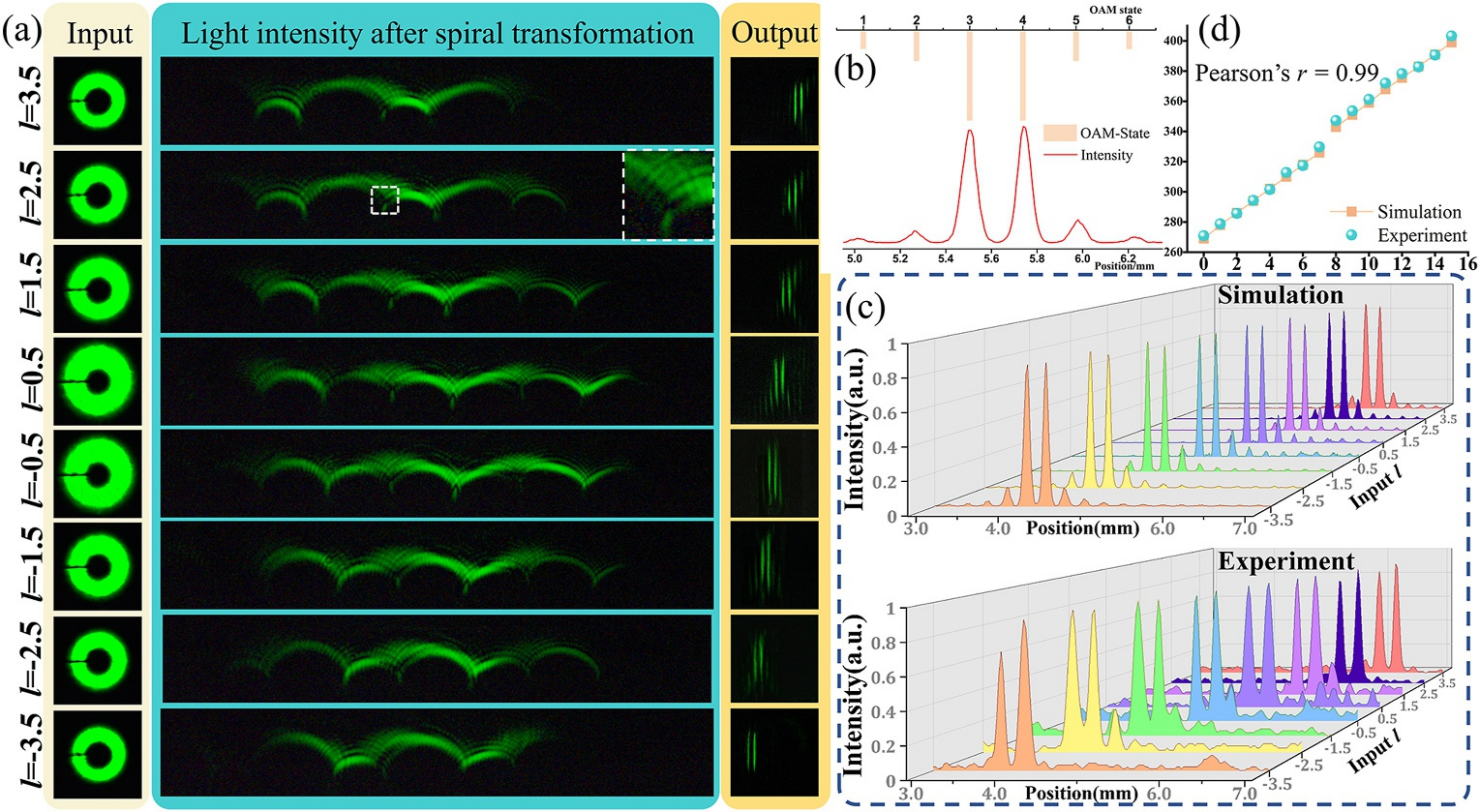
(a) Intensity distribution during the fractional OAM states sorting processing in the experiment. (b) Comparison between the one‐dimensional intensity distribution of the fractional OAM sorting and the corresponding OAM mode decomposition. (c) 3D characteristic peak maps for different TCs from both experimental and simulated results (More analysis see Supporting Information S1: Note 4). (d) Relationship between the characteristic peak positions (experimental and simulated) and the encoded symbols.

To explore the potential application of the proposed OAM states sorting method, that is, wind spiral transformation, as a proof‐of‐concept demonstration, we perform OAM‐assisted image encoding/decoding to functionally validate the proposed wind‐spiral sorter for structured‐light information transfer. Hexadecimal representation provides a compact and efficient means of data encoding, which is particularly advantageous in optical information processing [38]. As illustrated in Figure 6a, optical vortex array representations of a pixel with grayscale value 128 are shown in decimal (3 PLGs), binary (8 PLGs), and hexadecimal (2 PLGs) formats, respectively. The hexadecimal format has more concise information presentation capabilities. Because complete encoding of all grayscale values can be achieved only using one 2‐bit hexadecimal representation, we adopt this 2‐bit hexadecimal encoding scheme for grayscale image processing. As shown in Figure 6b, the left Encoding part shows a PLG beam array for hexadecimal encoding, with each TC corresponding to a different code symbol. The right Decoding part shows the corresponding results of OAM sorting.

**FIGURE 6:**
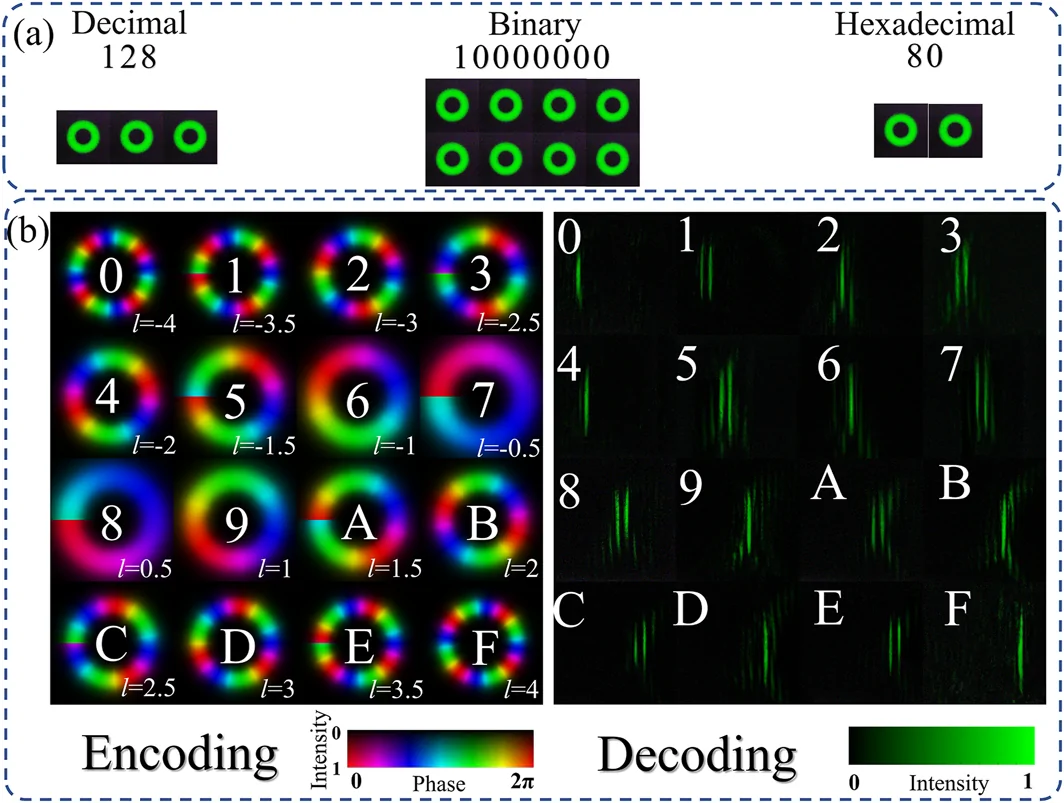
(a) Vortex array patterns corresponding to the grayscale value 128 represented in decimal, binary, and hexadecimal. (b) Encoding and decoding maps corresponding to the hexadecimal representation, respectively.

Because each pixel in a grayscale image has a grayscale value between 0 and 255, all pixel values can be encoded entirely using one 2‐bit hexadecimal representation. That is to say, grayscale values can be comprehensively represented using an optical vortex array consisting of two OAM states. Subsequently, to validate the theoretical framework described above, we performed grayscale image encoding and decoding experiments. We utilized a natural cloud vortex image of 372 × 407 pixels for the encoding demonstration, as shown in Figure 7a. First, a 5 × 2 pixel cell is randomly selected in the grayscale image, as shown in Figure 7a, and the corresponding grayscale values were extracted. After converting the extracted grayscale values into hexadecimal form, a 5 × 4 PLG beams array, as shown in Figure 7b, is generated based on the correspondence between hexadecimal characters and OAM states in Figure 6b. Then, the OAM states in this PLG beam array pass through the wind spiral transformation device in sequence, and decoding results were obtained, as shown in Figure 7c. The horizontal axis position of the characteristic peaks of the decoding results can be obtained by reading the one‐dimensional intensity profiles of the decoding results. Finally, these positions are mapped back to hexadecimal grayscale values to reconstruct the original image.

**FIGURE 7:**
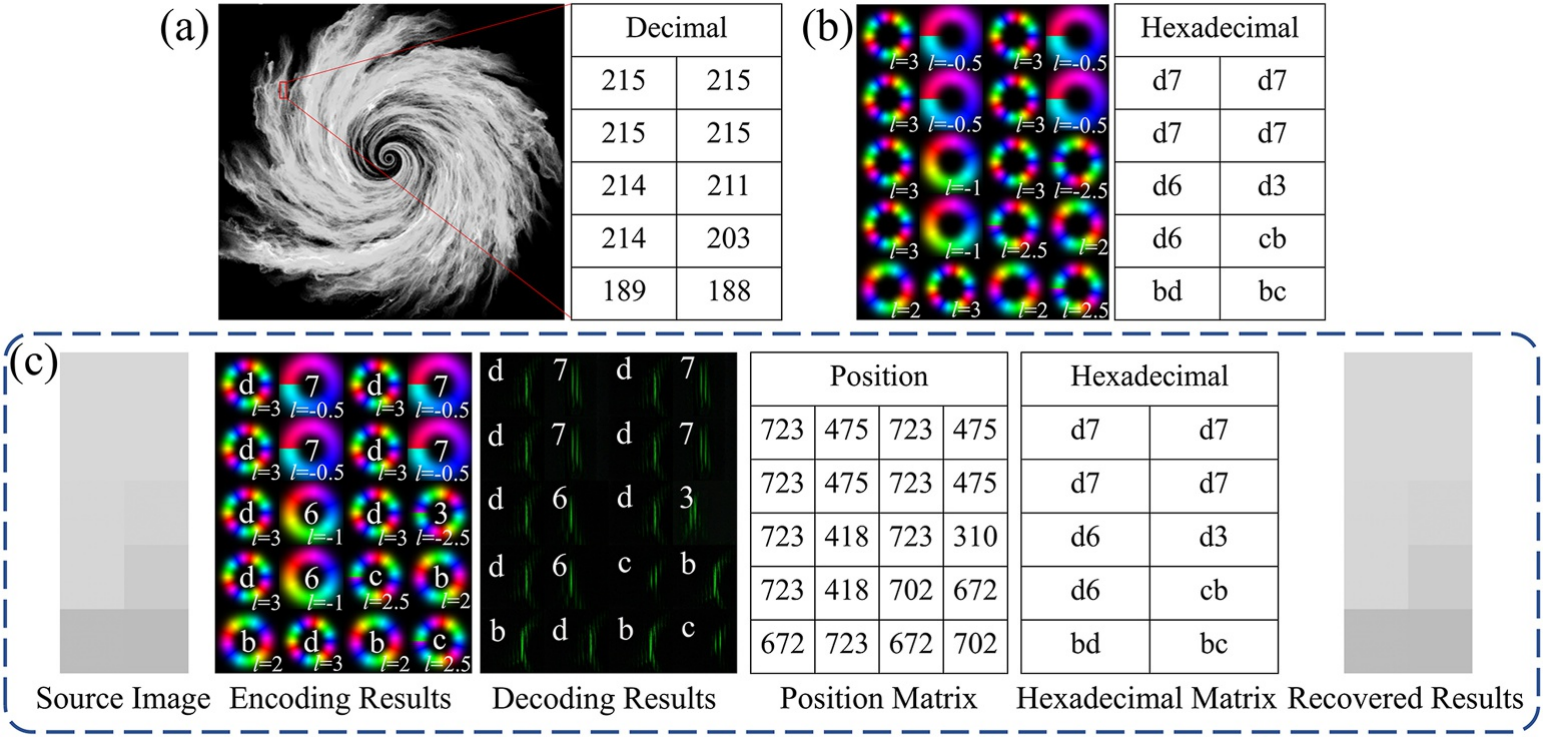
(a) Source image and grayscale values of the selected region. (b) Encoding the vortex array and the corresponding hexadecimal representation. (c) Each step of the encoding and decoding process was conducted from left column to right column.

At last, we conducted an experimental verification of the image encoding and decoding process using the natural cloud vortex image shown in Figure 7a as the source image. The detailed procedure is illustrated in Figure 8. First, the grayscale matrix of the source image is extracted, as shown in Figure 8b, and then converted into a hexadecimal grayscale matrix. Next, the hexadecimal grayscale matrix is encoded using PLG beams with different OAM states, generating a PLG beam array as shown in Figure 8c, where a pair of PLGs represented each byte. Subsequently, the PLG beam array was processed through the proposed wind spiral transformation for OAM sorting, and the sorting results are shown in Figure 8d. By reading the characteristic peaks' position, i.e., the bright fringes' horizontal position, from each decoded mode, we obtained the characteristic peak coordinate matrix, as illustrated in Figure 8e. Then, according to the previously established correspondence between coordinate positions and hexadecimal characters, the hexadecimal grayscale matrix was reconstructed, as shown in Figure 8f, and converted back into a decimal grayscale image. Finally, the source image was reconstructed from the grayscale matrix, as shown in Figure 8g, where pixels marked in red indicate decoding errors. According to the calculation, the bit error rate, which is defined by the ratio of the total number of encoded pixels (151,404 pixels) to the erroneous pixels (139 pixels), is as low as *P*_*e*_ = 0.09%. In case of developing commercial products, there are many conditions to be systematically considered, including capacity scaling, costs, latency, security implications, and so on. Furthermore, multiplexed FOAM can significantly improve encoding and decoding speed but it also introduces problems such as mode overlap and a lower signal background ratio (More details see Supporting Information S1: Notes 9 and 10).

**FIGURE 8:**
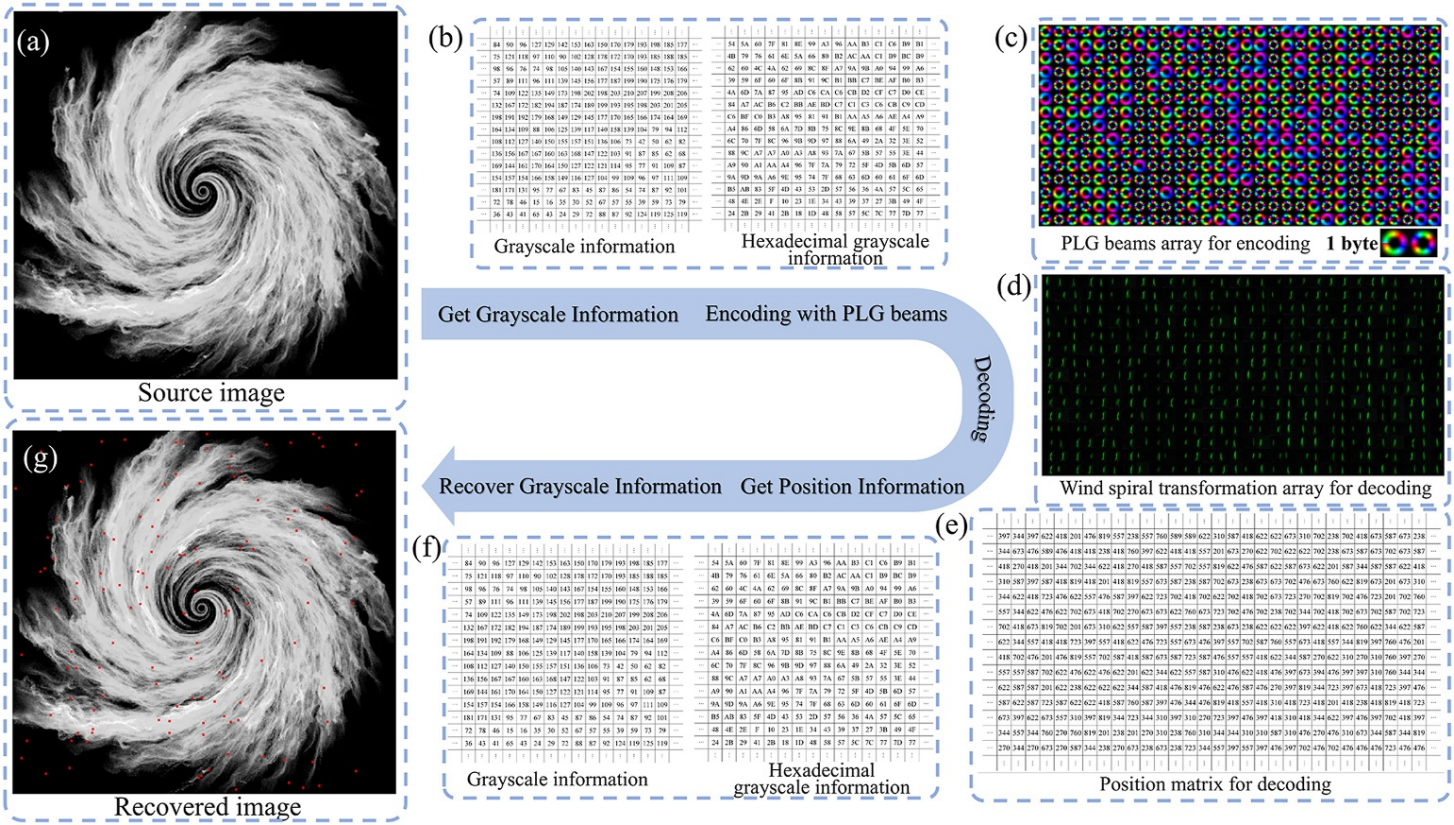
(a) Source image. (b) Grayscale information. (c) Vortex array for encoding. (d) Spiral transformation array for decoding. (e) Positions of characteristic peaks information. (f) Decoded grayscale information in decimal and hexadecimal formats, respectively. (g) Recovered image. See Supporting Information S1: Note 13 for the enlarged image.

## 4 Conclusions and Outlook

In summary, we have proposed an efficient and low‐crosstalk OAM sorting method, termed wind spiral transformation, which leverages fractional‐order OAM states to overcome the limitations on the number of sortable modes. Starting from the theoretical predictions, we propose a general spiral mode, that is, wind spiral transformation, which converts OVs with fractional OAM states into bright fringes at distinct spatial positions. Effective sorting is achieved by identifying the characteristic peak positions of these fringes. Numerical simulation analysis and experimental demonstrations validate the feasibility of sorting integer and fractional OAM states. Furthermore, the proposed OAM mode sorting technique is successfully implemented in optical image encoding and decoding, exhibiting high fidelity, with a measured bit error rate as low as 0.09%. It can be further reduced by increasing the channel range. Although the wind spiral transformation performs disadvantageous in FOAM multiplexing, it paves the way for optical communication based on FOAM and data storage system. In the future, this disadvantage may be solved by combining deep learning [39, 40]. More attractively, the proposed scheme can further provide encryption for the communication process, opening up new applications in both classical and quantum regimes.

## Author Contributions

**Xiaobin Yang:** writing – original draft, validation, methodology, investigation, conceptualization. **Mengran Fan:** investigation, validation, methodology, conceptualization. **Hao Zhang:** writing – review and editing, conceptualization, methodology, validation, investigation. **Kun Liu:** conceptualization, methodology, validation, investigation, writing – review and editing. **Juntao Hu:** writing – review and editing, conceptualization, investigation, validation, methodology. **Yuping Tai:** funding acquisition, writing – review and editing, resources. **Yijie Shen:** funding acquisition, writing – review and editing, resources. **Xinzhong Li:** funding acquisition, writing – review and editing, resources.

## Funding

The study was supported by the Natural Science Foundation of Henan Province (252300421485, 252300421770); the National Natural Science Foundation of China (12274116, 12447201,12404343); the State Key Laboratory of Transient Optics and Photonics, Chinese Academy of Sciences (SKLST202216); the Singapore Ministry of Education (MOE) AcRF Tier 1 grants (RG157/23 & RT1123); the Singapore Agency for Science, Technology, and Research (A*STAR) MTC Individual Research Grants (M24N7c0080), and the Nanyang Assistant Professorship Start Up grant.

## Conflicts of Interest

The authors declare no conflicts of interest.

## Supporting information


Supporting Information S1

## Data Availability

The data underlying the results presented in this paper are not publicly available at this time but may be obtained from the authors upon reasonable request.
