# Supplementary material for: Fractional Orbital Angular Momentum Sorting Using Wind Spiral Transformation
Source: Nanophotonics. 2026 Apr 17;15(8):e70086. doi: 10.1002/nap2.70086 (PMC13642060; doi:10.1002/nap2.70086)
Supplement: Supplementary file 1 [file NAP2-15-e70086-s001.docx]

**Fractional Orbital Angular Momentum Sorting using Wind Spiral Transformation: Supplementary document**

Xiaobin Yang^1^, Mengran Fan^1^, Hao Zhang^1^, Kun Liu^1^, Juntao Hu^1^, Yuping Tai^1,2,*^ Yijie Shen^3,4,*^ and Xinzhong Li^1,2,*^

*^1^School of Physics and Engineering & School of Chemistry and Chemical Engineering, Henan University of Science and Technology, Luoyang, 471023, China*

*^2^State Key Laboratory of Transient Optics and Photonics, Xi'an Institute of Optics and Precision Mechanics, Chinese Academy of Sciences, Xi'an, 710119, China*

*^3^Centre for Disruptive Photonic Technologies, School of Physical and Mathematical Sciences, Nanyang Technological University, Singapore*

*^4^School of Electrical and Electronic Engineering, Nanyang Technological University, Singapore*

*^*^*[*yptai@haust.edu.cn*](mailto:yptai@haust.edu.cn);[*yijie.shen@ntu.edu.sg*](mailto:yijie.shen@ntu.edu.sg);[*xzli@haust.edu.cn*](mailto:xzli@haust.edu.cn)

**This file includes:**

Supplementary Note 1. The conception of Wind Spiral.

Supplementary Note 2. Detailed derivation process of the spiral transformation existence conditions.

Supplementary Note 3. Experimental intensity patterns and corresponding experimental phase with different topological charge, both integer and fractional OAM.

Supplementary Note 4. The separation ratio and crosstalk matrix of OAM sorter based on wind spiral coordinate transformation.

Supplementary Note 5. Propagation characteristics of different precision fractional vortices in phase distribution.

Supplementary Note 6. The sorting results of the general fractional OAM states.

Supplementary Note 7. The impact of fractional OAM rotation on sorting results.

Supplementary Note 8. The impact of angular misalignment caused by input rotation on sorting results.

Supplementary Note 9. Multiplexed mode and the corresponding sorting results.

Supplementary Note 10. Multiplexed FOAM with overlap and corresponding sorting results.

Supplementary Note 11. The energy loss of different coordinate transformations.

Supplementary Note 12. The effect of different parameters of the wind spiral.

Supplementary Note 13. Enlarged version of the sub-image in Fig. 8 for reading convenience.

**Supplementary Note 1. The conception of Wind Spiral.**

The trajectory of an aircraft inspires the concept of the wind spiral. When an aircraft flying at a speed of ***v*_1_** encounters a ground wind with speed ***v*_2_**, its resultant velocity becomes ***v*_3_** = ***v*_1_** + ***v*_2._** The movement trajectory is influenced by the right triangle formed by airspeed, wind direction, and ground speed on the movement trajectory, as shown in Fig. S1.

Fig. S1 Schematic for wind spiral. *ρ* is the polar diameter and *θ* is the polar angle. ***v*_1_**, ***v*_2_**, and ***v*_3_** are ground, wind, and aircraft speeds. *r* is the basic cycle radius, *r*' is the offset distance from the basic cycle.

The expression of the wind spiral can be obtained after simple geometric calculations combined with the cosine theorem.

 (S1)

If *φ* = 0, equation (S1) can be simplified to the following form,

$\rho=r(\frac{v_{2}}{v_{1}}\theta+1)$ (S2)

This means that the aircraft moves forward and performs circular motion simultaneously; that is, the trajectory of the aircraft is an Archimedean spiral. If *φ* = π/2, equation (S1) can be rewritten as follows,

$\rho=r\sqrt{(\frac{v_{2}}{v_{1}}\theta)^{2}+1}$ (S3)

This means that the aircraft's moving direction is perpendicular to the radius of the basic circle, and the distance from the center of the circle changes with the arc, that is, the aircraft's trajectory is an involute spiral.

**Supplementary Note 2. Detailed derivation process of the spiral transformation existence conditions.**

Fig. S2 Schematic for coordinate transformation.

Figure S2 shows schematically a simple system to map any point (*x*, *y*) in plane P1 to a point (*u*, *v*) in plane P2. Consider a plane wave *ψ*_0_ = exp[i*φ*(*x*,*y*,*z*)], incident on plane P1, with direction cosines α, β, γ, giving *φ*(*x*,*y*,*z*) = *k*(α*x*+β*y*+γ*z*). Assuming normal incidence of *ψ*_0_ on plane P1, *ψ*_0_ = exp(i*k*z). If the phase *Q*(*x*, *y*) causes this mapping, the wave *ψ*_1_(x,y,z) leaving plane P1 is given by *ψ*_1_ = exp[*iφ*_1_(*x*, *y*, *z*)], where *φ*_1_ = *Q*(*x*,*y*) + *k*z = *k*(α_1_*x*+β_1_*y*+γ_1_*z*).

Then, from the above equations, we can get a partial differential equation.

, (S4)

where α_1_, β_1_, γ_1_ are direction cosines leaving plane P1. According to geometric optics, the following expression can be obtained easily,

$\left\{\begin{matrix} \alpha_{1}=\cos\theta_{x}=\frac{u-x}{r} \\ \beta_{1}=\cos\theta_{y}=\frac{v-y}{r} \end{matrix}\right.$, (S5)

where *r* = [(*u*-*x*)^2^+(*v*-*y*)^2^+*d*^2^]. Assuming the small-angle approximation *d* >> (*u*-*x*), (*v*-*y*), so *r* ≈ *d*. Combining equations (S4) and (S5), we get a part of the partial differential equations for *Q*(*x*, *y*),

$\left\{\begin{matrix} \frac{\partial Q(x,y)}{\partial x}=k\frac{u-x}{d} \\ \frac{\partial Q(x,y)}{\partial y}=k\frac{v-y}{d} \end{matrix}\right.$ (S6)

Next, we talk about how to solve the above partial differential equations. If ∂^2^*Q*(*x*, *y*)/ ∂*x*∂*y* and ∂^2^*Q*(*x*, *y*)/ ∂*y*∂*x* exist and are continuous, the differentiation order is interchangeable. Thus, a sufficient, but not necessary condition for equation (S6) to be solvable is that,

$\frac{\partial^{2}Q(x,y)}{\partial x\partial y}=\frac{\partial^{2}Q(x,y)}{\partial y\partial x}$ (S7)

Then, taking equation (S6) into equation (S7), we get the following equation,

$\frac{\partial u}{\partial s}\frac{\partial s}{\partial y}+\frac{\partial u}{\partial\theta}\frac{\partial\theta}{\partial y}=\frac{dv}{ds}\frac{\partial s}{\partial x}$ (S8)

For OAM sorting, the phase gradient along the azimuth in P1 should be mapped to a constant phase gradient along the *u* direction in P2; *u* and *v* show a linear relationship. Here, we have assumed that *u*(*θ*) is only related to *θ* and *v*(*s*, *θ*) is related to (*s*, *θ*). According to the Cartesian-polar transform and the above assumptions, equation (S8) can be further rewritten and organized in polar coordinates as,

$[u'(\theta)r_{s}-v_{s}r]\cos\theta+[v_{\theta}-v_{s}r_{\theta}]\sin\theta=0$ (S9)

This is the general condition on the spiral transformation (Uniqueness is not of concern here). Different types of spirals, *r* = *r*(*s*, *θ*), lead to different solutions. Here, we select the wind spiral, which is expressed in polar coordinates as *r* = *s*[1+(a*θ*)^2^]^1/2^, as the spiral transformation mode. For simplicity and demonstration, we set the coefficients of both trigonometric functions to zero, then the following expression can be obtained,

$\left\{\begin{matrix} u_{\theta}=\beta\\ v_{s}=\beta\frac{1}{s} \\ v_{\theta}=\frac{\beta a^{2}\theta}{1+a^{2}\theta^{2}} \end{matrix}\right.$ (S10)

and the solution of equation (S10) can be solved as,

$\left\{\begin{matrix} u=\beta\theta\\ v=\beta\ln(\frac{r}{r_{0}}) \end{matrix}\right.$ (S11)

Taking equation (S10) into equation (S6), two partial derivatives of the transform phase *Q* can be obtained,

$\left\{\begin{matrix} Q_{x}=\frac{k\beta}{d}\theta-\frac{kx}{d} \\ Q_{y}=\frac{k\beta}{d}[\ln(\frac{r}{r_{0}})]-\frac{ky}{d} \end{matrix}\right.$ (S12)

Finally, the transform phase *Q* can be written as the following expression,

$Q(x,y)=\frac{k\beta}{d}[\theta x+y\ln(\frac{r}{r_{0}})-y]-\frac{k(x^{2}+y^{2})}{2d}$ (S13)

However, this transformation introduces local angular tilts and non-paraxial components, causing the output beam to deviate from the optical axis. The corrective phase *P*(*u*,*v*) is necessary to correct these deviations and restore paraxiality. The following relation can be obtained by inversely deriving the relationship between the spiral parameters and coordinate (*u*, *v*), and substituting it into the sufficient, but not necessary, condition for the analytical solution of *P*.

$\left\{\begin{matrix} P_{\text{u}}=\frac{\text{k}}{d}\left[r_{0}\exp\left(\frac{v}{\beta}\right)\cos\left(\frac{u}{\beta}\right)-u\right] \\ P_{v}=\frac{\text{k}}{d}\left[r_{0}\exp\left(\frac{v}{\beta}\right)\sin\left(\frac{u}{\beta}\right)-v\right] \end{matrix}\right.$ (S14)

*P_x_* and *P_y_* are the partial derivatives of *P* with respect to *x* and *y*, respectively. Then, solving the system of partial derivatives equations, the analytical solution of *P* is as follows,

$P=\frac{k\beta r_{0}}{d}\exp\left(\frac{v}{\beta}\right)\sin\left(\frac{u}{\beta}\right)-\frac{k\left(u^{2}+v^{2}\right)}{2d}$ (S15)

**Supplementary Note 3. Experimental intensity patterns and corresponding experimental phase with different topological charge, both integer and fractional OAM.**

Fig. S3 (a1) − (a16) Experimental intensity patterns with different topological charge (TC). (b1) − (b16) Experimental interference patterns between the PLG intensity and a plane wave. (c1) − (c16) Retrieved experimental phase from the interference patterns.

In OAM sorting experiments, PLG beams with TCs ranging from −4 to 4 are used as input beams for sorting integer-order OAM states, while those with TCs from −3.5 to 3.5 are used for the sorting of fractional-order OAM states. The corresponding experimental results are illustrated in Figs. S3 (a1) - (a16), which are PLG beams carrying integer-order and fractional-order OAM states, respectively. Among them, Figures S3 (a9) - (a16) are PLG beams carrying fractional-order OAM states, where the intensity distributions exhibit distinct gap-like features, primarily caused by phase mismatch due to discontinuities. However, this does not significantly affect the accuracy of OAM mode sorting. Figures S3 (b1) – (b16) are the corresponding experimental interference patterns with a plane wave. Figure S3 (b9) – (b16) are the interference patterns of PLG beams with fractional-order OAM states. The mismatch of the interference fringes at the gap indicates phase discontinuities. Figures S3 (c1) – (c16) correspond to experimentally retrieved phase distributions obtained from the interference patterns.

**Supplementary Note 4. The separation ratio and crosstalk matrix of OAM sorter based on wind spiral coordinate transformation.**

Fig. S4. The performance metrics. (a) Integer OAM crosstalk matrix. (b) Fractional OAM crosstalk matrix. (c) Separation ratio (SR) of integer OAM sorter. (d) Separation ratio of fractional OAM sorter.

**Supplementary Note 5. Propagation characteristics of different precision fractional vortices in phase distribution.**

Fig. S5. Propagation characteristics of different precision fractional vortices in phase distribution. (a1) – (a5) The intensity of different FOAM charges (*l*) with different propagated distances (*D*_prop_). (b1) - (b5) The corresponding phase distribution. (c1) - (c5) The corresponding one-dimensional phase distribution along the circular paths in (b1) – (b5). *θ*_circ_ is the azimuth of the circular path.

During free space propagation, a fractional vortex collapses into a superposition of integer vortices. The fractional part of the fractional vortex loses its inherent property, which refers to the phase jump at the gap, after a certain distance. The relevant simulation results are shown in the following figure. When the fractional charge is set as 2.01, which represents the case where the fractional charge is accurate to two decimal places, the beam loses its fractional vortex inherent property after propagating 1mm. This means the fractional vortex with *l* = 2.01 does not have a precise topological charge after propagating 1mm. The fractional charge is set as *l* = 2.10, which represents the case where the fractional charge is accurate to one decimal place. For *l* = 2.10 and *D*_prop_ = 1mm, there is a phase jump at *θ*_circ_ = π. However, when the propagating distance reaches 300mm, the phase jump at *θ*_circ_ = π has disappeared. This means that the beams have collapsed into two integer vortices. For the case of a half-integer (e.g., *l* = 2.50), the phase jump still exists when the propagation distance reaches 2000mm. The maximum propagating distance of a half-integer fractional vortex is 4600mm. This is the reason that we chose half-integer OAM modes as information carriers.

**Supplementary Note 6. The sorting results of the general fractional OAM states.**

Fig. S6. The input beams with a fractional charge accurate to one decimal place and sorting results. (a1) – (a6) The input beams with a fractional charge accurate to one decimal place. (b1) - (b6) The corresponding sorting results. (c1) - (c6) The one-dimensional intensity distribution along the red line in (b1) - (b6).

To investigate the sorting capability of the wind spiral coordinate transformation for other fractional OAMs. The input beam's topological charge is set as a range from *l* = 2 to *l* = 3. As shown in Fig. S6, the sorting peak position gradually shifts to *l* = 3 as the fractional charge changes from *l* = 2 to *l* = 3.

**Supplementary Note 7. The impact of fractional OAM rotation on sorting results.**

Fig. S7. The impact of the input fractional vortex beam rotation on sorting results. (a1) - (a7) The input fractional vortex *l* = 2.5 with different angular rotations. *θ*_rota_ is the angular rotated. The white line marks the gap in the fractional vortex. (b1) - (b7) The corresponding sorting results. (c1) - (c7) One-dimensional intensity distribution along the red line in (b1) - (b7).

Considering the symmetry of the vortex, we simulated rotation of the fractional vortex gap from 0° to 180°. The one-dimensional intensity distribution along the red line shows periodic variations. When *l* = 2.5 and *θ*_rota_ = 0°, the sorting result of fractional vortex appears as two similar peaks. As *θ*_rota_ increases, the peak at *l* = 3 increases while the peak at *l* = 2 decreases, and the peak at *l* = 3 reaches its maximum and the peak at *l* = 2 reaches its minimum when *θ*_rota_ = 90°.

**Supplementary Note 8. The impact of angular misalignment caused by input rotation on sorting results.**

Fig. S8. The effect of the input fractional vortex rotation errors on sorting results. (a) The amplitude and phase of the input fractional vortex. (b) The one-dimensional intensity distribution with a little angular rotation.

The angular rotation of the input beams is injected as an error factor. Then, we calculate the one-dimensional intensity distribution at the same position. The results show that the rotation of the fractional vortex has no clear effect on the peak positions, but it does affect the amplitude of the peak. As shown in Fig. S8, the gray represents the error caused by the angular rotation of the input fractional vortex, while the blue line shows the one-dimensional intensity distribution without any angular rotation. The maximum error rate in peak amplitude is 8%, and the average error rate is 6.3%. This demonstrates that the method exhibits strong robustness against angular rotation.

**Supplementary Note 9. Multiplexed mode and the corresponding sorting results.**

Fig. S9. Multiplexed mode and the corresponding sorting results. (a1) – (e1) Single integer OAM mode, multiplexed integer mode, and the corresponding simulated (d1) and experimental (e1) sorting results. *l*_1_ is the topological charge of the first OAM mode, and *l*_2_ is the topological charge of the second OAM mode. (a2) – (e2) Fractional OAM mode, multiplexed fractional mode, and simulated (d2) and experimental (e2) sorting results. (a3) – (e3) and (a4) – (e4) Multiplexed mode combined integer and fractional mode, respectively.

To demonstrate the performance of the wind spiral transformation in multiplexed mode, the results of multiplexing the integer and fractional modes are provided. The results demonstrate that the background noise in the sorting results increases because of the interaction between different modes. Here, we performed simulated and experimental results using integer mode, fractional mode, and a combination of integer and fractional modes, respectively. To quantify the background noise, the signal background ratio (SBR) is introduced, which is defined as follows,

$$SBR=10*\log_{10}(\frac{\overline{P}_{signal}}{\overline{P}_{leakage}})$$

where $\overline{P}_{signal}$ is the average of signal power, and $\overline{P}_{leakage}$ is the average of the other region power. Using the sorting results of a single mode as a reference, the SBR of the single mode with *l* = 2 is calculated as 24.1 dB. Therefore, the interactions between multiplexed modes leads to increased background. One effective solution is combined with deep learning/artificial intelligence to filter out results with high background noise.

Deep learning is an excellent tool for exploring uncertain relationships. In the future, this limitation may be solved by deep learning, which has a special ability to find uncertain relationships. In our case, the sorting results of different adjacent FOAM modes are used as the training dataset. After training, a model for classifying adjacent FOAM modes is obtained. When a multiplexed FOAM sorting result with overlap is input, the output will show the components of the sorting result. However, to achieve optimal performance, a large amount of training data is required, and performance parameters must be continuously optimized.

**Supplementary Note 10. Multiplexed FOAM with overlap and corresponding sorting results.**

The results show modal overlap when the input is set to multiplexed mode. Due to the multiplexed mode, the overlapping position may exhibit constructive or destructive interference, as shown in Fig. S10. When the multiplexed FOAM is *l* = 2.5 and *l* = 3.5, the destructive interference exists at the position *l* = 3. On the contrary, when the multiplexed FOAM is *l* = 1.5 and *l* = 2.5, constructive interference occurs at position *l* = 2. Due to the interference, the peak position at *l* = 2 occurs a slight deviation when the multiplexed mode is *l* = 1.5 and *l* = 2.5.

Fig. S10. Multiplexed OAM sorting results with overlapping focal points. (a1) – (a3) Single fractional OAM mode and the multiplexed OAM modes. *l*_1_ is the topological charge of the first OAM mode, *l*_2_ is the topological charge of the second OAM mode. (b1) – (b3) The corresponding sorting results of (a1) – (a3). (c1) - (c3) Single fractional OAM mode and the multiplexed OAM modes. (d1) - (d3) The corresponding sorting results of (c1) – (c3). (e) The one-dimensional intensity distribution of the multiplexed OAM sorting result. (f) The one-dimensional intensity distribution of the multiplexed OAM sorting result.

**Supplementary Note 11. The energy loss of different coordinate transformations.**

Under the same experimental conditions, we measured the energy loss of the conventional coordinate transformation. The relevant results are listed in the following Table 1.

Table 1. The energy loss of different coordinate transformations. The Converter represents the energy utilization rate of distinct coordinate transformations. The Corrector represents the energy utilization rate of distinct coordinate transformations.

The results indicate that the energy loss of the OAM sorter based on coordinate transformations is approximately 95%. However, high energy loss does not affect to distinguish the horizontal positions of the characteristic peaks. In the future, energy loss can be reduced by using SLMs with lower energy loss or by fabricating corresponding micro/nano devices.

**Supplementary Note 12. The effect of different parameters of the wind spiral.**

Different parameters of the spiral in the wind spiral transformation affect the sorting results FWHM. The wind spiral is controlled by two parameters: *r*_0_ and a. The parameter “*r*_0_” controls the radius of the wind spiral, which is related to the radius of the input. The parameter “a” controls the space between adjacent spiral turns. It affects the sorting results by changing the number of spiral turns, as shown in Fig. S11.

Fig. S11. The effect of parameter “a” on sorting results. (a1) - (a4) Schematic diagram of the wind spiral parameter. (b1) - (b4) The corresponding sorting results with different parameters of wind spiral.

*θ_max_* is the azimuthal angle when the spiral radius equals *r*_max_. *N*_period =_ *θ_max_*/2π, which represents the number of spiral turns in the same vortex. As “a” decreases, the number of wind spiral turns increases significantly under the same vortex width, leading to an increase in OF. However, there is an upper limit to the increase in the OF. When *N*_period_ is set sufficiently high, the characteristic peaks disappear in sorting results. This is because the sorting results are mutually influential, and the FWHM of the characteristic peak is extremely small, presenting a speckle pattern.

**Supplementary Note 13. Enlarged version of the sub-image in Fig. 8 for reading convenience.**

Fig. S12 Enlarged image of the sub-image Fig. 8(b) in the primary document.

Fig. S13 Enlarged image of the sub-image Fig. 8(c) in the primary document.

Fig. S14 Enlarged image of the sub-image Fig. 8(d) in the primary document.

Fig. S15 Enlarged image of the sub-image Fig. 8(e) in the primary document.

Fig. S16 Enlarged image of the sub-image Fig. 8(f) in the primary document.
